# Targeting Mitochondrial Oxidative Phosphorylation Eradicates Acute Myeloid Leukemic Stem Cells

**DOI:** 10.3389/fonc.2022.899502

**Published:** 2022-04-29

**Authors:** Meixi Peng, Yongxiu Huang, Ling Zhang, Xueya Zhao, Yu Hou

**Affiliations:** ^1^ Biology Science Institutes, Chongqing Medical University, Chongqing, China; ^2^ Clinical Hematology, Third Military Medical University (Army Medical University), Chongqing, China; ^3^ School of Medicine, Chongqing University, Chongqing, China; ^4^ Key Laboratory of Laboratory Medical Diagnostics Designated by the Ministry of Education, School of Laboratory Medicine, Chongqing Medical University, Chongqing, China

**Keywords:** leukemic stem cells (LSCs), oxidative phosphorylation (OXPHOS), electron transport chain, tricarboxylic acid cycle (TCA cycle), mitochondria

## Abstract

Acute myeloid leukemia (AML) is a heterogeneous hematologic malignancy characterized by multiple cytogenetic and molecular abnormalities, with a very poor prognosis. Current treatments for AML often fail to eliminate leukemic stem cells (LSCs), which perpetuate the disease. LSCs exhibit a unique metabolic profile, especially dependent on oxidative phosphorylation (OXPHOS) for energy production. Whereas, normal hematopoietic stem cells (HSCs) and leukemic blasts rely on glycolysis for adenosine triphosphate (ATP) production. Thus, understanding the regulation of OXPHOS in LSCs may offer effective targets for developing clinical therapies in AML. This review summarizes these studies with a focus on the regulation of the electron transport chain (ETC) and tricarboxylic acid (TCA) cycle in OXPHOS and discusses potential therapies for eliminating LSCs.

## Introduction

Acute myeloid leukemia (AML) is a common type of acute leukemia with high lethality. Although conventional chemotherapy induces remission in up to 70% of AML patients, the majority of patients will relapse (1, 2). Leukemic stem cells (LSCs), originating from the hematopoietic stem cells (HSCs) and progenitor cells (multipotent progenitor cells, MPPs; common myeloid progenitor cells, CMPs), have self-renewal and infinite proliferative capacities and could block the function and development of mature blood cells and initiate leukemia when transplanted into a new host (3–6). Additionally, LSCs are resilient to the cytotoxic effects of chemotherapy *via* mechanisms that include resistance to apoptosis, increased capacities to efflux drugs, and relative quiescence (7, 8). Thus, the main treatment strategy for AML is the removal of LSCs to promote hematopoietic recovery and reduce recurrence.

In recent years, plenty of strategies have been developed to eliminate LSCs, such as blocking self-renewal pathways (9, 10), disturbing some signaling pathways (11–13), etc. However, these strategies show limited potential for curing AML and removing LSCs in clinical practice. Researchers are committed to finding new targets for eliminating LSCs. Metabolism reprogramming, especially energy metabolism, is considered a hallmark of cancer. In particular, energy metabolism may be the Achilles’ heel of cancer stem cells (14). To find an effective target for clearing LSCs, an increasing number of researchers are focused on finding the distinctive feature of energy metabolism. Lagadinou et al. revealed that LSCs relied on oxidative phosphorylation (OXPHOS) significantly more than normal bone marrow progenitors (15). Also, Kuntz et al. found that LSCs increased mitochondrial respiration and tricarboxylic acid (TCA) cycle flux compared with their counterparts (16). These studies indicate that LSCs depend on OXPHOS for survival, and understanding the metabolic properties of OXPHOS and further developing effective therapies to eradicate LSCs are required to improve the prognosis in AML patients.

We discussed here the metabolic properties of OXPHOS in LSCs.

## LSCs Depend on OXPHOS for Energy Demand

Mammalian cells produce adenosine triphosphate (ATP), the universal energy currency, mainly through glycolysis and OXPHOS. OXPHOS is the preferred method of energy production in mature cells with more metabolic efficiency than glycolysis, as glucose produces 38 ATP molecules *via* OXPHOS and two ATP molecules *via* glycolysis (17). Since the twentieth century, researchers have found that cancer cells, including both solid tumors and hematological malignancies (16, 18), preferred to depend on glycolysis for ATP production (19). However, several drug candidates targeting glycolysis in leukemic cells do not have a significant effect on tumor growth as expected. In 2018, Jones et al. performed global metabolic profiling in LSCs and AML blasts from 15 primary AML specimens by mass spectrometry and found that 39 metabolites were significantly increased in LSCs. Among the 39 metabolites, two were TCA cycle intermediates, and five were glutathione homeostasis metabolites, which were all related to OXPHOS (20). In 2020, Raffel et al. performed quantitative proteomics of LSCs and hematopoietic stem and progenitor cells (HSPCs) and found that the predominant utilization of OXPHOS and not glycolysis may be a general feature of LSCs (21). Also, many other studies disclosed that tumors are metabolically heterogeneous and LSCs paradoxically depend more on OXPHOS to meet their energy demands and maintain their survival (15, 22, 23), which may be the reason that drug candidates targeting tumor glycolysis sometimes lead to cancer treatment failure. Moreover, HSCs tend to be glycolytic for survival. Thus, targeting OXPHOS may kill LSCs effectively.

OXPHOS, a fundamental mitochondrial process, is carried out by the electron transport chain (ETC) to the production of ATP. In more detail, the metabolites NADH and FADH2, mainly from the TCA cycle, could fuel the complex in ETC and contribute to the proton-motive force, which drives the ATP synthesis. Thus, OXPHOS is generally considered to link the TCA cycle and ETC (24). As the metabolites from glucose, amino acids, and fatty acids can enter the TCA cycle, OXPHOS generates energy not only *via* glucose metabolism but also through amino acid and fatty acid metabolism. Many researchers are working on exploring the specific metabolic regulation of LSCs. In 2012, Lagadinou et al. found that the mitochondrial oxidative metabolism was activated in LSCs. Also, they used the mitochondrial respiration inhibitors oligomycin and FCCP to treat LSCs and found that the cells failed to activate the glycolytic pathway to satisfy energy demand even though OXPHOS was inhibited (15). Thus, LSCs are metabolically inflexible and cannot depend on glycolysis for energy demand, which leads to glucose no longer being considered a fuel for OXPHOS. As a result, LSCs use nutrients, amino acids, and/or fatty acids to fuel OXPHOS. This discovery was also confirmed by Jones et al. in 2018 (20).

The metabolic properties of OXPHOS in LSCs vary in different stages of disease pathogenesis. The *de novo* AML LSCs use amino acid metabolism to fuel OXPHOS. It has been reported that some specific amino acids, such as glutamine, cysteine, and branched-chain amino acids, are important in many hematologic malignancies, but the mechanism remains unknown (25–27). Until 2018, Jones et al. revealed that LSCs isolated from *de novo* AML patients showed increased amino acid levels and uptake. Moreover, targeting amino acid metabolism decreased TCA cycle flux and induced LSC death (20). Whereas, both fatty acid oxidation (FAO) and amino acid metabolism are important to drive OXPHOS in relapsed LSCs. Stevens et al. reported that relapsed LSCs were resistant to amino acid metabolism inhibition, and the inhibition of both amino acid metabolism and FAO led to a decreased OXPHOS and eliminated LSCs more clearly than one inhibition (23, 28). The difference in metabolism properties between *de novo* AML LSCs and relapsed AML LSCs may represent potential targets for individualized treatment.

There are many factors influencing OXPHOS in LSCs, and these factors may present potential targets for eliminating LSCs.

## Regulation of ETC in LSCs

### Composition of ETC

**Figure 1:**
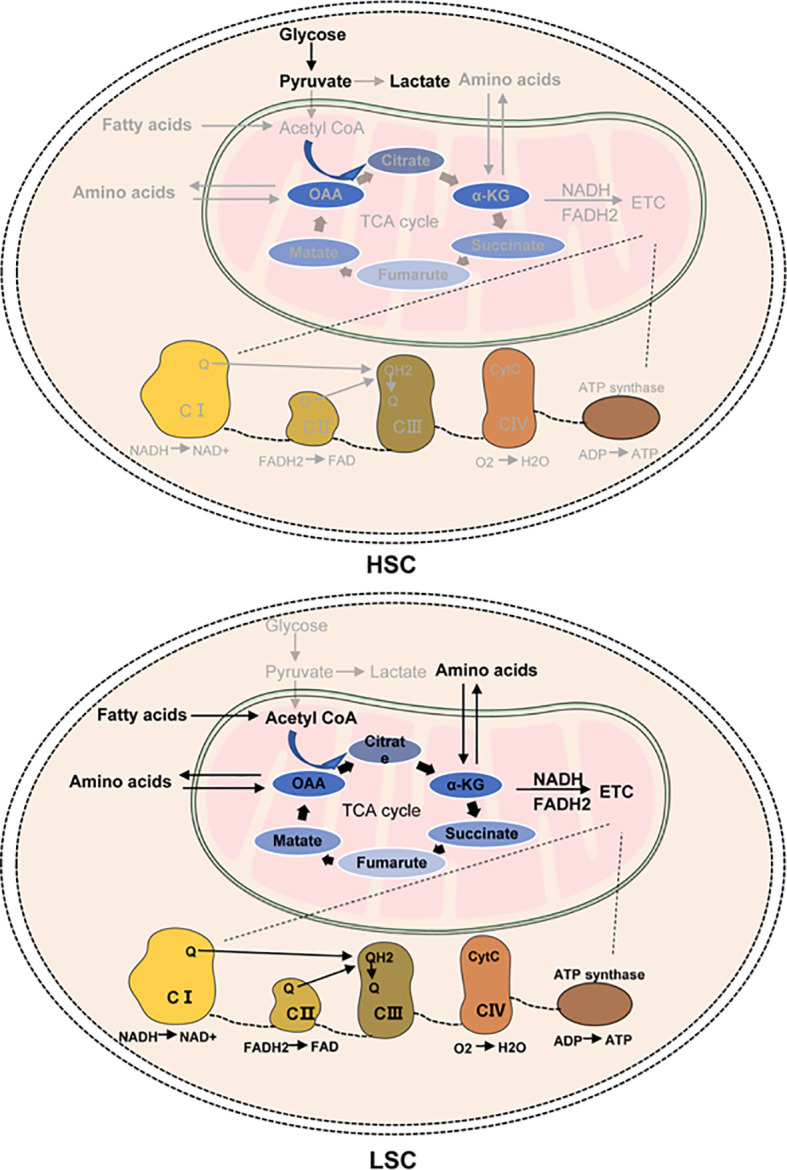
The energy metabolism in HSCs and LSCs. LSCs mainly depend on OXPHOS for energy demand. OXPHOS is a fundamental mitochondrial process, linking the tricarboxylic acid (TCA) cycle to the production of ATP *via* the electron transport chain (ETC). In LSCs, glucose cannot enter the TCA cycle. Amino acids enter the TCA cycle through conversion to the TCA cycle intermediates (such as oxaloacetate (OAA) and alphaketoglutarate (α-KG)). Fatty acids are converted to acetyl-CoA through β-oxidation in the mitochondria. The increased NAD+ levels from nicotinamide metabolism are necessary for the sufficient function of the TCA cycle enzymes. NADH and FADH2 produced from the TCA cycle generate ATP *via* ETC. TCA, tricarboxylic acid; ATP, adenosine triphosphate; acetyl-CoA, acetyl coenzyme A; CytC, cytochrome c; ETC, electron transport chain; Q, coenzyme Q/ubiquinone; QH2, ubiquinol; FADH2, flavin adenine dinucleotide; NADH, nicotinamide adenine dinucleotide; CI, complex I; CII, complex II; CIII, complex III; CIV, complex IV; ADP, adenosine diphosphate.

ETC is composed of four major multienzymatic complexes and supported by two mobile cosubstrates (coenzyme Q and cytochrome c) in the inner mitochondrial membrane. As shown in **Figure 1**, nutrient oxidation can produce NADH and FADH2, the electron donor molecules that are also called high-energy-reducing equivalents, mainly from the TCA cycle. NADH2 provides electrons to the NADH dehydrogenase:ubiquinone oxidoreductase (complex I (CI)), and FADH2 transfers electrons to the succinate:ubiquinone oxidoreductase (complex II (CII)). Coenzyme Q uptakes the electrons from CI and CII and transfers them to the ubiquinol:cytochrome c oxidoreductase or cytochrome bc1 complex (complex III (CIII)). Cytochrome c then uptakes the electrons from CIII and transfers them to cytochrome c oxidase (complex IV), which transfers the electrons to molecular oxygen. Ultimately, ATPs are produced at the ATP synthase with the help of the electrochemical gradient caused by a proton pump from the mitochondrial matrix to the intermembrane space (29–32).

### Mitochondrial-Encoded Translation of ETC Proteins in LSCs

There are about 90 proteins in ETC, and 13 of these are encoded by mitochondrial DNA, which is composed of a double-stranded circular genome without introns. Mitochondrial DNA-encoded proteins are translated into mitochondrial ribosomes using a unique protein translation machinery, differing from nucleus DNA-encoded proteins, which are translated into cytosolic ribosomes. Although most ETC-related proteins are obtained from cytoplasmic translation, most of these proteins are not located in the key sites of ETC. Also, cytoplasmic translation has a wide range of functions. Thus, it is not a good choice to intervene in ETC *via* targeting cytoplasmic translation (33). Inversely, most of the 13 mitochondrial-encoded proteins are functional components of 4/5 ETC complexes, and targeting mitochondrial-encoded translation can inhibit the activity of ETC effectively (34). In 2011, Skrtic et al. found that antimicrobial tigecycline, an inhibitor of mitochondrial protein translation, selectively killed LSCs rather than their normal counterparts *via* inhibiting OXPHOS (33). Importantly, antimicrobial tigecycline is an FDA-approved agent. While Jhas et al. reported that the metabolic shift with decreased OXPHOS resulting from antimicrobial tigecycline was reversible upon removal, the long-term use of antimicrobial tigecycline treatment may cause clinical resistance (35). In 2017, Liyanage et al. revealed that inhibiting the replication of mitochondrial DNA by ddC (a selective inhibitor of the mitochondrial DNA polymerase) repressed ETC-related proteins and induced cell death in both subsets of primary leukemic cells and LSCs (36). Also, there was no toxicity of ddC for normal cells in xenotransplantation assays. However, it remains unknown whether ddC could be effective in patients. Thus, mitochondrial-translated proteins may be a potential target for LSCs, while the effective treatment strategy targeting them to eliminate LSCs needs further study.

### Posttranslational Modifications of ETC Proteins in LSCs

Glutathionylation, one of the posttranslational modifications, has a critical role in sustaining the activity of OXPHOS (37, 38). For example, glutathionylation of hMIA40, an intermembrane space import receptor of mitochondria, maintains the optimum function of CIII and CIV in ETC (39). Also, Jones et al. revealed that glutathionylation of succinate dehydrogenase complex flavoprotein subunit A (SDHA), a key component of ETC CII, enhanced OXPHOS in LSCs (40). Cysteine is the precursor of glutathione in LSCs. Inhibition of SDHA glutathionylation *via* cysteine depletion or using cysteine-degrading enzyme impairs ETC CII activity and OXPHOS and leads to LSC death with no detectable effect on HSCs (40). However, the glutathionylation of ETC proteins is reversible and can be removed by potent reducing agents (such as β-mercaptoethanol) as detected in HEK293T cells, indicating that long-term clinical use of some of these potent reducing agents may cause resistance. Thus, to explore more effective targets, more studies should focus on the mechanism of glutathionylation in ETC-related proteins. In 2020, van Gastel et al. found that residual mouse AML cells in the bone marrow required glutamine to fuel glutathione synthesis and generate pyrimidine nucleotides, but not the TCA cycle (41), and whether enhanced glutathione synthesis promotes OXPHOS *via* ETC-related protein glutathionylation remains unknown.

Other posttranslational modifications of ETC exist, such as phosphorylation of the complex I subunits (42, 43), methylation of the NDUFB3 subunit in complex I (44), acetylation of the complex II subunits (45), etc. However, whether these posttranslational modifications of ETC exist in LSCs remains unknown.

### Mitochondrial Protease Activity Regulating the ETC Complex in LSCs

Mitochondrial proteases, including four core ATP-dependent proteases (m-AAA, i-AAA, LonP, and ClpXP), regulate protein homeostasis in the mitochondrial membrane and matrix. m-AAA is encoded by AFG3L2, and AFG3L2 mutation or knockdown leads to unfolded mitochondrial protein accumulation and complex IV deficiency, resulting in respiratory deficiency mainly in neurological diseases (46, 47). i-AAA is encoded by YME1L, and the knockdown of YME1L by short hairpin RNA leads to excessive accumulation of non-assembled respiratory chain subunits and impairs cell proliferation in human embryonic kidney 293 cells (48), whereas the function of i-AAA is not found in other cells. As for LonP, there is no related research about its function. ClpXP, containing serine protease ClpP and the AAA+ATPase C1pX (49), is the most important protease to maintain protein quality in mitochondria *via* degrading damaged or misfolded substrates involved in ETC and the TCA cycle (50–53). Moreover, ClpP is highly expressed in LSCs and is tight with ETC activity and cell function. The knockdown of ClpP reduces the growth and viability of AML progenitors *via* degrading SDHA and impairing complex II activity (50). Moreover, A2-32-01, a ClpP inhibitor, decreases the number of leukemic cells in primary and secondary engraftment in mice (33). Ishizawa et al. revealed that ONC201 and ONC212, activators of ClpP, also decreased OXPHOS in AML through uncontrolled degradation of respiratory chain proteins in complexes I, II, and IV (54). Moreover, ONC201 is being studied in phase I clinical trials, and ONC212 has shown promising preclinical results in AML (55–57). Thus, these studies show that not only inhibition but also hyperactivation of ClpP impair the ETC activity and induce cell death, and whether inhibiting or activating ClpP is the preferred therapeutic strategy of eradicating LSCs remains unknown.

Additionally, neurolysin (NLN) is a zinc metallopeptidase with secretion into the circulation or location in the mitochondria. The function of NLN in the circulation has been well studied, while the mitochondrial function is unclear. Until 2020, Mirali et al. found that NLN was necessary for the formation of respiratory complexes I, III, and IV in AML cells (58). 3-[(2S)-1-[(3R)-3-(2-Chlorophenyl)-2-(2-fluorophenyl)pyrazolidin-1-yl]-1-oxopropan-2-yl]-1-(adamantan-2-yl)urea (R2), an inhibitor of NLN, reduces the viability of LSCs *in vitro* and *in vivo*, but does not influence normal hematopoietic cells. Whether NLN inhibitor could be used in the clinic and if there are other effective targets relating to mitochondrial proteases in the removal of LSCs, need further study.

### Other Factors Regulating ETC

FAO supplies FADH2 to ETC. Cytosolic fatty acids could convert into fatty acyl-CoA esters by acyl-CoA synthases, and the fatty acyl-CoA is transported to the mitochondria to perform FAO, thus resulting in FADH2 production. This metabolism signaling indicates that FAO could fuel ETC directly, not through the TCA cycle, but whether this metabolism pathway exists in LSCs remains unknown.

Also, there are some other strategies targeting ETC in LSCs to treat AML: IACS-010759 (59) and mubritinib (60) have been used to inhibit complex I and ME-344 to inhibit complexes I and III (61). Though the mechanisms remain unclear, IACS-010759, mubritinib, and ME-344 have achieved a good result in clearing LSCs *in vitro* or *in vivo*. Notably, these treatments do not influence the survival of normal stem and progenitor cells, suggesting that these therapies may be more selective for LSCs. Furthermore, IACS-010759 is currently in phase I clinical trials in R/R AML (62). A ribonucleoside analog, 8-chloro-adenosine (8-Cl-Ado), can remove LSCs while sparing HSC *via* targeting the ATP synthase. Also, 8-Cl-Ado has been used in phase I clinical trials and has yielded encouraging results in AML patients (23).

## Regulation of the TCA Cycle in LSCs

### Glycolysis as Fuels to the TCA Cycle

Not only can glucose be oxidized to produce ATP and regenerate NADH, it can also enter the TCA cycle through glycolysis. In 2019, Jones et al. revealed that the AML blast cells were dependent on glycolysis to produce energy and upon glucose deprivation to decrease viability in culture. Glucose depletion did not affect the viability or colony-forming potential of LSCs, which indicated that LSCs are not dependent on glucose to fuel the TCA cycle and survival (20). In the same year, Chu et al. reported that six1 played a role in LSC maintenance in AML, and six1-KD led to a significant decrease in the glycolytic gene expression and pyruvate and lactate production, but did not affect the ATP level (63). This research indicated that glycolysis was important for LSC maintenance but not through energy suppling. In 2020, Pulikkottil and Bamezai revealed that quiescent LSCs preferentially depended on OXPHOS, while non-quiescent, cycling AML LSCs may likely depend on glucose metabolism (64). Also, Zhao et al. reported that the hypoxic endosteal niche led to LSCs undergoing a metabolic change (abandoning OXPHOS and switching to glycolysis) in chronic myeloid leukemia (65). Whether the metabolic change exists in LSCs of AML is unclear. Thus, further study of glycolysis in AML LSCs is needed.

Fructose is the most abundant blood sugar in humans apart from glucose. In 2016, Chen et al. found that AML cells were prone to fructose utilization to fuel glycolytic flux and result in increased proliferation and colony growth when glucose was deficient (66). As colony formation is linked to cancer stem cell properties (67), the increased fructose utilization may contribute to maintaining LSCs. While further study about fructose metabolism in LSCs was needed.

### PPP as Fuels to the TCA Cycle

Glucose is involved in the pentose phosphate pathway (PPP) to generate pentose phosphate for ribonucleotide synthesis, serine biosynthesis, and one-carbon metabolism (68). The metabolites of PPP can also enter glycolysis *via* glyceraldehyde 3-phosphate and fructose 6-phosphate. As reported, PPP had a critical role in leukemic cell survival, and the suppression of PPP enzymes by shRNA-mediated knockdown or inhibitor selectively decreased leukemogenesis *in vitro* (69, 70). In particular, Mizuno et al. found that EVI1 can both upregulate the PPP pathway and OXPHOS in AML (70). Also, Pei et al. reported that PPP was a cytoprotective mechanism of AML cells in response to the treatment of parthenolide (71). Thus, whether the PPP pathway exists in LSCs and is related to glycolysis or the TCA cycle need further study.

### Amino Acid Metabolism as Fuels to the TCA Cycle in LSCs

Glutamate is the key metabolite in the process of amino acid metabolism fueling the TCA cycle. It was reported that arginine, glutamine, histidine, branched-chain amino acids, and proline were precursors to glutamate. Glutamate could enter the TCA cycle by converting to α-ketoglutarate with the help of glutamate dehydrogenase and drive OXPHOS through the production of NADH and succinate (the substrate for ETC complex II) (72). Jones et al. revealed that deprivation of amino acids could decrease fumarate, malate, citrate, succinate, and α-ketoglutarate in the TCA cycle and induce the death of *de novo* AML LSCs, but not HSCs, indicating that *de novo* AML LSCs use amino acid metabolism to fuel the TCA cycle and OXPHOS (20). Moreover, the pharmacological inhibition of amino acid metabolism with a combination of venetoclax and azacytidine (ven/aza) reduces OXPHOS and eliminates LSCs by measuring the engraftment ability of primary AML specimens after 24 h of *ex vivo* treatment (20). Notably, in 2018, FDA approved venetoclax as an AML treatment agent in combination with other hypomethylating agents. Thus, amino acid metabolism may be a potential target to treat AML patients in the clinic.

### FAO as Fuels to the TCA Cycle in LSCs

FAO, a process of breaking down fatty acids into acetyl-CoA, fuels the TCA cycle. Within the mitochondria, fatty acyl-CoA enters the FAO spiral resulting in the generation of acetyl-CoA that contributes to the overall mitochondrial pool due to acetyl-CoA being used in the TCA cycle (**Figure 1**). The relapsed AML patients have inferior outcomes compared to the *de novo* AML patients after receiving ven/aza treatment, which may be due to FAO also fueling the TCA cycle (73). Shafat et al. revealed that the AML blasts could alter the metabolic processes of adipocytes to activate lipolysis, and then the fatty acids were transferred from adipocytes to the AML blasts and sustained AML proliferation in the bone marrow microenvironment (74). Also, leukemic cells could create an inflammatory microenvironment in adipose tissue, triggering lipolysis. More importantly, the released fatty acids fuel the chemoresistance of LSCs by inducing the fatty acid transporter CD36 expression (75). Interestingly, the inhibition of fatty acid synthesis has also been shown to potentiate the effects of the BCL-2/BCL-XL dual inhibitor, ABT-737, in AML (76). Inhibiting carnitine palmitoyltransferase 1a (CPT1a), the rate-limiting enzyme of the essential step of FAO, enhances the antileukemic activity of Bcl-2 inhibitor in AML (77, 78). Also, in relapsed LSCs, sorbitan sesquioleate, a CD36 inhibitor to inhibit fatty acid uptake, in combination with ven/aza, results in a significant decrease in OXPHOS (20, 28). Thus, the inhibition of FAO and its role in fueling OXPHOS will likely become an exciting new direction in fighting therapeutic resistance in AML.

### Other Metabolism Pathways as Fuels to the TCA Cycle in LSCs

In addition to amino acid metabolism and FAO fueling the TCA cycle and OXPHOS, other metabolisms exist. For example, Anderson et al. revealed that dihydrolipoamide *S*-succinyltransferase (DLST), a TCA cycle-related transferase, was important for the TCA cycle flux and MYC-mediated leukemogenesis of T-cell lymphoblastic leukemia (79). Anderson et al. also found that heme export was closely related to the TCA cycle and OXPHOS (80). Whether these regulations exist in the LSCs remains unknown. In 2020, Jones *et al.* revealed that elevated NAD+ from nicotinamide drives OXPHOS by regulating NAD+-dependent enzymes (isocitrate dehydrogenase, 2-oxoglutarate dehydrogenase, and malate dehydrogenase) in the TCA cycle in relapsed LSCs (81). Notably, the inhibition of nicotinamide phosphoribosyltransferase (NAMPT), the rate-limiting enzyme of nicotinamide, converted to NAD+ in mammals, decreased the TCA cycle flux, and enhanced ven/aza sensibility. Thus, the finding provides a means for LSCs to circumvent the cytotoxic effects of ven/aza therapy and suggests that inhibition of NAMPT may be an effective strategy to target LSCs in relapsed AML patients.

## Resistance to OXPHOS Inhibition

Based on the important role of OXPHOS in LSCs, many therapies targeting OXPHOS have been used to eliminate LSCs. However, none of them can erase LSCs. In 2021, Saito et al. found that OXPHOS inhibition triggered a mitochondrial transfer from the mesenchymal stem cells (MSCs) to AML cells *via* tunneling nanotubes, which induced mitochondrial fission and increased functional mitochondria in AML cells, leading to secondary resistance to OXPHOS inhibition (82). Thus, interference with these processes is required to boost the antitumor potency of OXPHOS inhibition. In 2022, You et al. reported that AML cells took up functional mitochondria from MSCs to sustain the resistance to cytarabine (83). Thus, targeting mitochondrial transfer in a leukemic microenvironment may be an effective strategy for curing AML, and an effective target needs further study.

Also, some noncanonical energy fuels exist in leukemic cells. Creatine, as a noncanonical energy fuel, can satisfy the cellular energy requirement in an acute manner coupled with the ATP–ADP transition when a nutrient deficiency occurs. Creatine-dependent metabolism was found important for the survival of EVI1-positive AML and FLT3-ITD mutant AML (84, 85). Whether creatine metabolism contributes to LSC survival is unclear and needs further exploration.

## Conclusions

The crucial role of LSCs in the progression and relapse of AML has resulted to investigations into the LSC unique metabolic profile, which may help develop novel and effective treatment strategies. OXPHOS may be a potential target for AML treatment. In this review, we discussed OXPHOS in LSCs, focusing especially on the regulation of the TCA cycle and ETC (**Figure 1**) and the intervention strategies targeting OXPHOS (**Table 1**). However, several questions and perspectives remain to be considered in this field. The dysregulated metabolism and the metabolic changes after OXPHOS inhibition in LSCs should be further characterized. In addition, toxicities from OXPHOS-targeted therapies should be evaluated as metabolism is a ubiquitous process.

**Table 1:** Modulators of OXPHOS in LSCs.

Drugs	Category	Target	Reference
Antimicrobial tigecycline	FDA-approved	ETC-related protein translation	(11, 27, 29)
ddC	–	ETC-related protein translation	(30)
A2-32-01	–	ClpP	(27)
ONC201	Phase I clinical	ClpP	(50, 51)
ONC212	–	ClpP	(52)
R2		NLN	(53)
Cysteine-degrading enzyme	–	Complex II	(34)
IACS-010759	Phase I clinical	Complex I	(33, 56)
Mubritinib	–	Complex I	(54)
ME-344	–	Complexes I and III	(55)
8-Cl-Ado	–	ATP synthase	(17)
Venetoclax	FDA-approved	BCL2 and DNA methylation	(17, 23, 63, 69)
ST1326	–	FAO	(65, 66)
Sorbitan sesquioleate	–	CD36	(23, 62)
Inhibiting NAMPT	–	Nicotinamide metabolism	(69)

## Data Availability Statement

The original contributions presented in the study are included in the article/supplementary material. Further inquiries can be directed to the corresponding author.

## Author Contributions

MP reviewed the literature and wrote the manuscript. YoH reviewed the literature. YuH, XZ, and LZ designed the review and revised the manuscript. All authors listed have made a substantial, direct, and intellectual contribution to the work and approved it for publication

## Funding

This work was supported by grants from the National Natural Science Foundation of China (Grant No. 82170115 and No. 1900588).

## Conflict of Interest

The authors declare that the research was conducted in the absence of any commercial or financial relationships that could be construed as a potential conflict of interest.

## Publisher’s Note

All claims expressed in this article are solely those of the authors and do not necessarily represent those of their affiliated organizations, or those of the publisher, the editors and the reviewers. Any product that may be evaluated in this article, or claim that may be made by its manufacturer, is not guaranteed or endorsed by the publisher.
